# Supplementary material for: Are carbon emissions trading and green financial instruments synergistic? -Comprehensive quantitative research based on content analysis
Source: PLoS One. 2024 Mar 7;19(3):e0298601. doi: 10.1371/journal.pone.0298601 (PMC10919601; doi:10.1371/journal.pone.0298601)
Supplement: S1 Data — (ZIP) [file pone.0298601.s001.zip › Data/地级市Z_F工作报告.zip!/╜¡╦╒/╦▐╟¿╩╨/└┤╫╘╣1⁄2╓┌║┼í╛┬φ┐╦╩2╛▌═°í┐_2019.docx]

各位代表：

　　现在，我代表市人民政府向大会作工作报告，请予审议，并请市政协各位委员和其他列席同志提出意见。

2018年政府工作回顾

　　过去的一年，我们在省委、省政府和市委的坚强领导下，在市人大、市政协的监督支持下，始终以习近平新时代中国特色社会主义思想为指导，深入贯彻党的十九大精神，紧紧围绕高质量发展要求，坚持稳中求进工作总基调，积极践行新发展理念，深入推进供给侧结构性改革，坚决打好“三大攻坚战”，全力推动“六增六强”，经济社会保持平稳健康发展，主要经济指标保持在合理区间。预计全年地区生产总值增长6.5%，一般公共预算收入增长2.8%，固定资产投资增长7%，社会消费品零售总额增长8%，全体居民人均可支配收入增长9.3%。

　　（一）招商引资实现新突破。始终把招商引资摆在“头版头条”，全力以赴推进招大引强。着力完善招商体系。创建“168”招商体系，成立北京、上海、浙江等异地宿迁商会，构建“三位一体”招商机制，制定重点产业链招商导则和产业图谱，招商引资组织化、专业化水平明显提升。积极组织招商活动。持续开展招商引资“百日攻坚”活动，成功举办中国宿迁绿色产业洽谈会，先后在市外开展140场招商推介活动。全年符合开工条件的亿元以上工业项目达306个，计划投资1090亿元，当年新引进10亿元以上重大项目14个、增长55.6%。

　　（二）项目建设取得新成效。牢固树立“项目为王”理念，坚定不移谋项目、上项目。创新项目建设机制，成立重大产业、重点工程、民生实事3个工作领导小组，强化项目建设的统筹、调度和推进工作，坚持月通报、季观摩和常态性督查，确保项目建设稳步实施。22项为民办实事项目顺利完成；197个重大产业项目建设达到序时进度，实现投资703.2亿元；142项中心城市重点基础设施项目全面推进，实现投资256亿元。

　　（三）产业升级迈出新步伐。坚持在做大总量中调优结构、在提升质量中加快升级，全力推动产业发展转向高质量。工业经济量质齐升。规上工业增加值增长7.5%，实现工业开票销售收入、工业入库税收2521亿元、139.1亿元，分别增长20.2%、24.9%，增速均位居全省前列，工业规模进一步壮大。大力实施千百亿级产业培育工程，产业发展呈现“三高一降”，即机电装备、纺织服装和食品饮料三大重点产业产值、新兴产业产值、高新技术产业产值占工业总产值比重达57.8%、11%、26.5%，分别提高10.3个、2个、2.5个百分点，高耗能行业产值占比下降0.8个百分点，工业结构进一步优化。深入推进“521”工程，新增开票销售超2000万元企业410家、超亿元企业92家，新增挂牌上市企业15家，纳税百强企业销售收入增长27.5%，企业质效进一步提升。现代农业加速发展。持续推进农业结构调整，新增生态高效农业面积26.1万亩，设施农业、设施渔业占比分别提升至18.56%、31.1%。新增高标准农田35.92万亩，农业机械化水平达83%，成功获批国家农业科技园区，农业现代化水平不断提高。积极培育新型经营主体，新增省级以上龙头企业9家，家庭农场数量居全省首位。“宿有千香”农产品区域公用品牌正式发布，新增绿色食品107个、地理标志农产品3个，农产品影响力不断提升。服务业规模持续壮大。服务业增加值增长6.5%左右。旅游业蓬勃发展，全年接待国内外游客2486万人次、实现旅游总收入293亿元，分别增长12%、15%。电子商务快速发展，电商筑梦小镇入选全国最美特色小镇50强，“淘宝村”“淘宝镇”数量分别位居全国第六位和第八位，全市网络零售额预计达290亿元、增长20.8%。金融服务能力持续增强，制造业贷款余额263亿元，增长7.3%、居苏北前列；企业直接融资占比达24%，提高1.74个百分点；着力化解重点企业资金链风险，金融风险总体可控；泗阳县、宿城区获评“省金融生态优秀县”。科技创新能力不断增强。净增国家高新技术企业37家，新增省级工程技术研究中心7个，新认定院士工作站3个。引进高层次人才及团队128个，16人入选省“双创人才”、居苏北第二。全市新申请专利、授权专利、企业专利申请和授权量增幅实现“四个全省第一”。

　　（四）精准脱贫增添新成果。以国家扶贫改革试验区建设为抓手，聚焦三大片区、低收入群体和经济薄弱村，深入实施精准脱贫攻坚工程。片区发展基础进一步夯实。基础设施、产业发展、民生改善三大类264个项目稳步实施，溧河洼特大桥、330省道等一批重大基础设施项目建设完工，26个农业产业示范项目建成运营，贫困地区发展内生动力持续增强。村级集体经济加快发展。引导村集体通过开发集体资源等形式创收，省定经济薄弱村集体经营性收入村均超过23.9万元、增长25.8%，46个省定薄弱村实现脱贫、累计脱贫率达79.8%。政策支撑效果明显。完善资产收益扶贫、健康扶贫、教育扶贫、保险扶贫等政策体系，惠及群众55.3余万人次。全市12.8万低收入人口实现脱贫，低收入人口发生率由15.1%下降至5.1%，全面完成年度脱贫任务。

　　（五）城乡建设呈现新面貌。着眼一体发展，大力推动城乡协调并进、融合共进。中心城市品质持续提升。创成省生态园林城市，以全省第一名通过全国水生态文明城市建设试点验收。着力改善市民生活环境，完成棚户区改造130万平方米、老旧小区改造33个，累计惠及群众7.6万人；16个街头绿地建成投用，9家单位庭院完成开放式改造，中心城市建成区绿地率、人均公园绿地面积分别提高到40.57%、15.47平方米；依法拆除中心城区48座高立柱广告。全面优化功能配套，完成双星大道、黄浦江路北延等12条道路改造，新改建停车场12个，新增停车泊位5000余个，完成50组公共场所充电桩建设、3350辆公共自行车更新升级，市民出行更加便捷；高标准建成城市之家5个，新改建菜市场9个，万达广场开业运营，建成现代公厕88座，市民生活更加方便；新铺设雨污管网60.1公里，雨污分流率达89.8%。实施文明城市长效建设十大专项行动，推行“宿迁文明20条”2.0版，市区社会文明程度测评指数保持全省前列；文明交通不断深化，市交警支队被公安部记集体一等功。县城建设联动推进。沭阳县成功入选“全国新型城镇化质量百强县”，土地节约集约利用工作得到国务院表彰。泗阳县创成国家生态文明建设示范县，高分通过全国文明城市提名城市测评。泗洪县“五城同创”扎实推进，通过省级优秀管理城市验收，获批省级食品安全示范县。乡村风貌有效改善。制定13个专项行动计划，全力推动乡村振兴。谋划并启动农民群众住房条件改善工作，完成农村危房改造2276户。实施村庄环境综合整治工程，着力抓好“点亮乡村”“四好农村路”建设，完成129条乡村道路路灯安装、总里程达274.8公里，新改建农村公路447公里、危桥108座，完成安全生命防护工程1460公里，农村道路交通事故死亡人数同比下降38%。

　　（六）生态建设有了新进展。深入践行“两山”理论，积极探索“生态优先、绿色发展”新路径。环境容量不断扩大。持续推进百万亩生态林建设，新造生态林10.7万亩，完成杨树更新改造26.9万亩。生态修复工作有序开展，建成30条生态示范河道，完成骆马湖“退渔还湖”2.07万亩、洪泽湖湿地修复超过1万亩。基础设施持续完善。新建改造城镇污水收集管网525.8公里，建成乡镇污水处理设施12个、村庄污水处理设施252个。完成10个乡镇垃圾分类运输体系建设，中心城区生活垃圾分类投放设施覆盖率、餐厨废弃物集中处理率分别达50%、85%。污染治理深入开展。全力抓好中央环保督察“回头看”反馈问题整改，妥善处理洪泽湖生态环境污染事件，持续实施“263”专项行动，PM2.5浓度、空气质量优良率改善幅度分别位居全省第四、第七；30条城市水体消除黑臭，“一线一岸”整治基本完成，12个县级以上集中式饮用水水源地、7个国考断面水质达标率均为100%。

　　（七）改革开放再上新台阶。坚持以改革增动力、以开放添活力，为高质量发展提供有力支撑。重点改革纵深推进。经济体制改革持续深化，不断拓展“放管服”改革深度，“证照分离”试点稳步推进，“信用承诺简化审批”做法在全省推广，获批全国“企业开办全程网上办”改革、全省首批“不见面审批”标准化等多项试点；持续推进资源要素价格改革，工商业用水和非居民用天然气销售价格全省最低；着力化解企业历史遗留问题，全面落实减税清费政策，工业企业收费项目全省最少；设立总规模45亿元的产业发展基金，着力解决企业融资需求。农业农村改革扎实开展，农村产权流转交易总额全省第二，农村“两权”抵押贷款试点业务规模位居全省前列；全年共转让土地增减挂钩节余指标2939.45亩、总量全省第一。社会事业改革不断深入，积极落实省委深化教育体制改革要求，新增普通高中招生计划1944人；出台支持和规范意见，推动社会办医疗机构高质量发展。载体支撑愈发有力。宿迁经开区、宿迁高新区、沭阳经开区功能配套日趋完善，主导产业加快集聚，泗阳、泗洪和宿城开发区综合实力稳步提升。6个南北共建园区在全省考核中再次领跑，苏宿园区连续9年名列全省第一。对外开放不断深化。积极引导企业参加进博会、广交会等境内外展会，预计进出口总额和出口总额分别增长18.7%、19.7%，增速均位居全省前列。全年新设外商投资企业94家、增长118%，实际利用外资3.76亿美元。新增国际友城3对，与3个城市新签署友好合作备忘录，友城交流合作愈发紧密。

　　（八）民生福祉得到新改善。聚焦群众所急所盼，积极办好民生实事，着力补齐民生短板。增收基础不断夯实。深入开展“春风行动”，大力推进“家门口就业”工程，全年新增转移农村劳动力3.21万人，城镇登记失业率1.81%、低于省定目标2.19个百分点。实施全民创业行动计划，新增全民创业项目3.1万个，带动就业超过8万人。社会事业日趋繁荣。中心城市基础教育布局调整有序推进，青海湖路小学、北大附属宿迁实验学校建成投用；全市高考本科达线率达73.42%，21人被清华北大录取、居苏北第一；职业教育技能大赛省赛金牌数及奖牌数均列苏北第二。乡镇医院能力提升工程深入实施，完成新一轮219家村居卫生机构提档升级；人均基本公共卫生服务经费补助标准提高到65元。农村无害化卫生户厕普及率达92.1%，区域供水入户率达95%。“时尚体育四季嘉年华”系列活动持续开展，十九届省运会金牌、奖牌和总分取得历史性突破，宿迁籍运动员周欣茹夺得青奥会摔跤项目金牌、胡道亮实现第三届亚残会参赛项目“大满贯”。“文化四季”等活动深入推进，完成359个基层综合文化服务中心、20个农村文化大院建设。兜底保障更加有力。企业职工“五险”新增34.6万人次，基本养老、基本医疗保险覆盖率均达96%以上，低收入农户养老和医疗保险参保率双双实现100%。农村“五保”、城乡孤弃儿童保障标准稳步提高，3541名残疾儿童享受基本康复免费服务。率先建立失能特困老人集中供养制度，被列为国家级居家和社区养老服务改革试点地区。社会治理成效明显。荣获全国城市信用建设创新奖和全国守信激励创新城市，“宿迁之声”微博荣获2018年江苏政务新媒体十大最具影响力奖，政府信息公开标准化建设获批全国唯一国家级试点项目。12345政府服务热线接听群众来电24.66万件，群众满意率达96.23%。法治政府建设满意度始终保持90%以上。安全生产事故数量和死亡人数继续实现双下降。治安问题有效解决率、矛盾纠纷调处化解率和平安创建活动群众参与率均居全省第一，信访形势平稳向好，社会大局和谐稳定。粮食、供销、供电、食药、气象、地震、民防、民族、宗教、侨务、人民武装和双拥、档案、党史、地方志等工作实现新进展，妇女、儿童、青少年、老龄、科协、红十字、慈善等事业取得新进步。

　　各位代表，去年以来，在宏观形势复杂严峻、困难挑战不断增多的情况下，我们始终坚持同心协力、接续奋斗，超前谋划、综合施策，牢牢把握发展主动权，巩固了稳定健康的发展势头、政通人和的社会大局。回首一年工作，我们深深体会到，做好政府工作：必须切实提高政治站位，以更高更快的行动自觉落实好中央和省委、省政府部署要求。以习近平同志为核心的党中央坚强领导，是我们应对各种挑战的定海神针。我们要始终坚持以习近平新时代中国特色社会主义思想为指导，自觉把“四个意识”、“两个维护”贯穿于政府工作始终，坚决贯彻落实党中央、国务院和省委、省政府工作要求，并与宿迁实际紧密结合，创造性地开展工作，确保落实部署不走样、把准方向不偏航。必须牢牢把握第一要务，以有力有序的项目建设筑牢稳中求进的发展根基。发展不充分、不平衡是宿迁当前最大的问题。我们要始终高举发展大旗，坚持“四化同步”，全力聚焦新型工业化、新型城镇化，按照系统化思维、项目化推进的理念，把发展任务细化为一个个项目，把项目建设分解到一个个节点，一件一件落实，一项一项推进，保持定力、扭住关键、精准发力，不断开创工作新局面。必须时刻紧扣群众诉求，以实实在在的民生福祉促进政风民心的同频共振。执政为民是政府一切工作的落脚点。我们要始终把群众呼声作为第一信号，时刻将群众安危冷暖挂在心头，将群众所急所盼所怨记在脑中；把群众利益作为第一选择，倾力解决好群众最关心最直接最现实的教育、医疗、饮食、出行等民生实事，让发展更有温度，让幸福更有质感；把群众满意作为第一标准，让人民评判、使人民满意，在全市上下形成同心同德干事业、同向同力谋发展的良好局面。必须保持担当有为状态，以“功成不必在我”的胸襟营造干事创业的浓厚氛围。一任有一任的责任，一任有一任的担当。我们要始终坚持干字当头，着力做好打基础、利长远的实事要事，全力攻坚关注多、影响广的急事难事，不贪一时之功，不图一时之名，求真务实、开拓创新、埋头苦干，始终保持力度不减、韧劲不松，努力交出一份人民满意、群众认可的成绩单。

　　各位代表，过去一年成绩来之不易，令人振奋！这些成绩的取得，是党中央、国务院正确指引的结果，是省委、省政府和市委坚强领导的结果，是全市人民团结拼搏、社会各界协同奋斗的结果。在此，我代表市人民政府，向奋战在各行各业的全体劳动者，向人大代表、政协委员，向各民主党派、工商联和无党派人士、各人民团体，向离退休老同志，向中央和省驻宿单位以及人民解放军、武警、公安干警，向参与宿迁建设的海内外客商、广大援宿干部，以及所有关心支持宿迁发展的各级领导、各界人士，致以崇高的敬意和衷心的感谢！

　　在肯定成绩的同时，我们也清醒地看到，全市经济社会发展中还存在一些矛盾和问题：经济下行压力加大，增长速度优势有所弱化；产业发展总量偏小、层次不高，转型升级任重道远；实体经济步履维艰，发展面临诸多挑战；城乡融合发展、群众住房条件亟需提升；环保问题仍然突出，生态环境质量改善任务艰巨；公共服务还有不少短板，民生工作与人民群众对美好生活的需求还有不小差距；政府职能转变有待加快、作风建设有待深化，等等。对此，我们一定高度重视，采取有力措施加以解决。

2019年政府工作目标

　　今年政府工作的主要预期目标是：地区生产总值增长6.5%以上，一般公共预算收入增长5%左右，规上工业增加值增长7.5%左右，固定资产投资增长7%左右，社会消费品零售总额增长8.5%，高新技术产业产值占规上工业产值比重达28%，全社会研发投入占GDP比重达1.7%，万人发明专利拥有量达2.78件，城镇登记失业率稳定在3%以内，全体居民人均可支配收入增长8.5%左右，基本完成农村低收入人口脱贫任务，节能减排和大气、水环境质量完成国家、省下达的目标任务。

　　在实现上述重要经济和社会指标的同时，今年政府将重点抓好以“三优三安四提升”为主要内容的100项民生实事：

　　一是持续推进教育布局优化。在中心城区规划建设20所中小学和幼儿园，加快缓解“入学难”“择校热”“大班额”问题，让更多的孩子享受到更加方便、更加公平、更加优质的教育。

　　二是持续推进医疗服务优质。建立健全医疗卫生服务体系，每个县区建设1-2所区域医疗卫生中心，让农村居民就近就地看得上病、看得起病、看得好病。

　　三是持续推进供水保障优良。加强集中式饮用水水源地长效管护，改造更新1500公里农村地区供水管网，让城乡居民喝上干净、卫生、优质的“放心水”。

　　四是着力推动人民群众安居。实现6.6万户农村居民住房条件改善，完成164万平方米中心城市棚户区改造，推动城市物业管理提升，让城乡居民住得更舒心，生活得更省心。

　　五是着力推动公共出行安心。实施好市区停车便利化、“好巴士”快速公交、智慧交通、公共自行车升级等项目，新建改造农村公路704公里，建成安全生命防护工程1545公里，进一步改善交通条件，让群众出行更便捷、更安全。

　　六是着力推动食药供给安全。实施校园“明厨亮灶”工程，提升学校食品安全管理水平，保障孩子们“舌尖”上的安全；加快农贸市场快速检测室建设，让群众吃得更放心、食品安全少操心。

　　七是提升收入水平。实施就业促进工程、创业扶持行动、妇女“家门口就业”、电商人才培训、职业技能培训等项目，新增城镇就业4.5万人，转移农村劳动力2.8万人，让更多的人创业有门路、就业有渠道、收入有保障、致富有途径。

　　八是提升帮扶成效。大力实施精准脱贫，将城乡低保标准提高到每人每月520元，推行低收入农户大病补充保险，切实保障好困难群体的基本生产生活；加强农村留守儿童和孤弃儿童关爱保护，力争让每一名孩子都能接受到良好教育，健康快乐成长。

　　九是提升服务能力。加快城市之家、农贸市场、城市书房建设和体育场馆开放，让市民享受到更贴心、更暖心的公共服务。

　　十是提升环境质量。启动中心城市西南片区水环境综合治理，完成15条城市黑臭水体整治；加快提升大气质量，努力让天更蓝、水更清、地更绿、景更美，城市更靓丽、农村更清新。

　　新的一年，各县区也将同步抓好共计401项的民生实事。我们将积极顺应人民群众对美好生活的向往，通过市县区联动，每年集中解决一批事关群众切身利益的问题，让改革发展成果更多更公平地惠及全体人民，让全市人民的生活一年更比一年好。

全力做好2019年政府重点工作

　　今年政府工作的指导思想是：坚持以习近平新时代中国特色社会主义思想为指导，深入贯彻党的十九大和十九届二中、三中全会精神，按照中央经济工作会议、省委十三届五次全会和市委五届七次全会工作部署，坚持稳中求进工作总基调，坚持新发展理念，坚持推动高质量发展，坚持以供给侧结构性改革为主线，立足建设“江苏生态大公园”发展定位，进一步增强“进”的信心、保持“稳”的定力、展示“新”的作为、强化“深”的拓展，继续打好“三大攻坚战”，认真做好“六个稳”工作，着力激发微观主体活力，推动高质量发展“六增六强”落地见效，为全面建成小康社会收官打下决定性基础，以优异成绩庆祝中华人民共和国成立70周年。

　　重点抓好以下七个方面的工作：

　　（一）聚力推进项目建设，全面增强发展动能。牢固树立“项目为王”理念，扎实开展“项目建设突破年”活动，全力以赴抓项目、增投入、促发展。

　　全力突破招商引资。进一步完善“168”招商体系，不断提升招商水平，致力实现“四量”突破。在项目数量上求突破。聚焦重点招商区域，深入开展产业链、资本链、创新链、人才链“四链”招商，引进亿元以上符合开工条件项目230个以上、3000万美元以上外资项目7个以上。在项目体量上求突破。紧盯国资央企、世界500强、民企100强、行业50强等，精准推介、定向攻关，引进10亿元以上符合开工条件项目30个。在项目质量上求突破。围绕投资强度、税收额度、科技高度、链条长度、环保程度、能耗限度“六度”标准，严把项目准入门槛，从源头上提升招商质量。在招商力量上求突破。组建武汉、重庆区域招商局，鼓励行业协会、商会参与招商。接续开展“百日攻坚”招商活动，精心筹备绿色产业洽谈会、台湾·宿迁周经贸合作恳谈会、台商走电商等系列活动，努力实现招商成效与招商氛围双提升。

　　深入推进“521”工程。实施规模发展行动。加大“小升规”企业培育力度，全年净增规模以上企业120家，新增开票销售收入超亿元企业70家以上。实施兼并重组行动。支持企业强强联合、兼并整合，完成出资额超500万元兼并重组企业70家。实施技术改造行动。引导企业“设备换芯”“生产换线”“机器换人”，完成设备投资超千万元技改项目330个。实施股改上市行动。引导企业对接多层次资本市场，新增挂牌上市企业17家。实施成果转化行动。拓宽科技成果转化通道，新增省级以上新产品新技术100个、转化高科技成果200项以上。

　　扎实抓好重大项目建设。突出重大产业项目建设。推进华东光谷、恒天化纤等三大类超200个、计划总投资超1800亿元重大产业项目建设，确保江林智能制造、桔家衣柜等141个项目投产达效，全年完成投资900亿元以上。突出重大基础设施项目建设。加快推进324省道、268省道等项目，实现徐宿淮盐高铁、综合客运枢纽建成投用，争取宿连高速开工建设，全年完成重大基础设施项目投资60亿元。做好合宿新高铁、宿连航道等重大项目前期准备工作，力争尽快开工。突出重大城建工程项目建设。抓好八大类170项中心城市重点基础设施项目建设，全年完成投资330亿元，迎宾大道、上海路等项目建成投用。

　　（二）聚力培育千亿产业，不断夯实经济支撑。按照“巩固、增强、提升、畅通”要求，持续深化供给侧结构性改革，进一步做大产业规模，做优产业质量，做强产业实力。

　　着力壮大工业制造业。重点加快产业“三化”进程：推动产业集群化。将打造重点产业链条作为千百亿级产业培育的关键抓手，加快构建“5+13+X”产业链体系，推动产业规模不断壮大，机电装备、食品饮料、纺织服装、家居制造、功能材料分别实现主营业务收入700亿元、660亿元、630亿元、370亿元、310亿元。各县区进一步明确产业主攻方向，加快打造一批超500亿级的重点产业集群。推动产业高端化。聚焦智能改造，加快关键工序、核心装备升级换代，实施智能化改造项目50个；聚焦新动能培育，加快推动工业互联网、大数据、云计算、人工智能与产业发展深度融合，实施企业信息化改造项目30个，创建星级“上云”企业15家以上。推动产业绿色化。深入实施能效提升、清洁生产等技术改造，加快淘汰落后产能，创建国家级绿色制造示范1个，工业固体废弃物综合利用率保持在95%以上，单位GDP能耗下降1%，单位GDP用水量下降6.3%。

　　着力培育现代服务业。大力实施服务业“三大行动计划”，加快构建“343”服务业新体系，力争服务业增加值增长11%以上。推动生产性服务业增容扩量。以专业化、体系化、集聚化为方向，加快发展现代物流、现代金融、科技服务三大生产性服务业，年内生产性服务业增加值占比提高3个百分点。做大现代物流产业，完善物流体系，实现物流业增加值120亿元。做强现代金融业，加强金融机构引培，加快保险小镇、基金小镇建设，年内金融业增加值达150亿元。推动生活性服务业拓展优化。以便利化、精细化、品质化为方向，提升现代商贸、现代旅游、健康养老、房地产四大生活性服务业，力争社会消费品零售总额突破900亿元。培植健康养老产业，启动三台山康养旅居项目建设，打造健康服务业示范园、健康养生基地。促进房地产业健康发展，加快城乡空间整理，科学把控土地供应规模节奏，切实稳定房地产市场预期。推动新兴服务业集聚发展。以规模化、示范化、高端化为方向，壮大电子商务、文化、信息服务三大新兴服务业。发展信息服务业，加快建设浪潮（苏北）大数据运营中心、京东云华东数据中心二期，培育一批物联网软件、系统集成和运营服务重点企业。发展文化产业，启动四大类、27个大运河文化带项目建设，打造文化创意、数字出版等产业集聚区。围绕建筑业超千亿目标，加快推进产业转型，扶持重点企业做大做强，年内培育施工总承包特级资质企业1家。

　　着力建设高层次发展平台。建好园区平台。以节约化、集约化为方向，推动宿迁经开区、沭阳经开区、宿迁高新区争先进位，支持泗阳开发区争创国家级经济技术开发区、泗洪开发区争创省级特色创新示范园区、宿城开发区争创国家生态工业示范园区。加大南北共建园区建设力度，确保苏宿园区实现考核“十连冠”。建好开放平台。加快运河宿迁港三期前期工作，实现二类水路开放口岸正式开通运行。完善综保区基础设施，努力推动综保区申创工作取得突破。推进跨境电商产业园建设，跨境电商进出口总额超过1亿美元。

　　着力提升科技创新能力。坚持科技创新第一动力，努力构建以企业为主体、市场为导向的创新体系。突出研发平台建设。围绕构建“3+5+2+X”科技创新平台，推动科技综合体、技术创新中心、协同创新中心等新型研发机构建设，力争全年新增省级工程研究中心5个以上。加快宿迁（上海）离岸研发基地建设，着力提升科研飞地贡献力。推进创新孵化平台建设，完善科技创业孵化链条，创建省级以上星创天地、众创空间各2家。强化创新主体培育。实施创新型企业“双百双千”培育工程，推动科技型中小企业、高新技术企业和瞪羚企业成长，鼓励企业加大研发投入，净增国家高新技术企业40家。鼓励柔性引才，引进高层次创新创业人才及团队100个以上。推动科技专项突破。瞄准产业发展瓶颈制约，组织实施重大科技专项技术攻关，加快突破关键技术，年内转化重大科技专项8个以上。

　　（三）聚力打造中国名城，加快提升城市能级。围绕建设“生态优美的湖滨城市、充满活力的创业城市、人民满意的宜居城市”定位，大力推进东、西、南、北、中“五大片区”建设，切实增强中心城市承载力和带动力。

　　打造水美城市。充分利用“两湖两河”的水资源优势，加快建设水、城、人一体发展的水美城市。推进湖城一体。推动滨湖城市发展，加快湖滨新区建设，统筹推进骆马湖岸线、大运河湿地公园、山水绿廊、上相湾等项目，着力把湖滨片区打造成水美城市的典范。推进城水交融。坚持把水韵充分融入城市，加快城市东西向河道建设，进一步优化城市河网系统；加强水泡布点，让城市拥有更多的水、更美的水。扎实推进河湖水系沟通工程，让水流起来、活起来。推进人水相亲。发挥“两河”贯城资源优势，实施古黄河风光带、大运河文化带景观提升工程，加快建设亲水项目，着力打造“两河”城市标识。系统推进城市水环境优化工程，扎实做好雨污管网完善、水系沟通、景观配套，启动西南片区水环境治理，加快西民便河疏浚等项目建设。

　　打造宜居城市。以国家生态园林城市、国家森林城市和联合国人居环境奖创建为抓手，着力提升城市“五度”：提升文明度。进一步巩固全国文明城市创建成果，深入推行“宿迁文明20条”2.0版，推进文明城市创建由市区向县城拓展，着力打造文明城市群，让文明有礼成为宿迁城市特质。提升舒适度。加强城市绿色廊道和重要节点绿化建设，积极推进青年公园、楚风公园、虞姬公园等项目，建成12处街头绿地和民生游园。加快建设“没有围墙的城市”，90%以上的机关单位实现“敞院”开放。有序实施28个城市棚户区和16个老旧小区改造，加快智慧物业建设，着力提高城市物业管理水平，进一步改善居住环境。启动中心城区排水防涝能力提升工程，完成10个易涝点改造、8条排涝河道疏浚。提升整洁度。持续开展环境卫生、街景容貌等治理，加强对各类城市家具的动态保洁和维护，着力打造“最干净城市”。聚焦群众反映强烈的突出问题，不断深化综合整治，推动城市秩序再上新台阶。提升便捷度。实施路网加密和道路快速化改造工程，完成黄河南路、青年路、金沙江路等道路改造，新建改造城市道路18.74公里，建成洪泽湖路、发展大道2条快速公交专用道。进一步完善公共服务设施，新建农贸市场5个、公共停车位5000个。优化提升社区商业、医疗等综合服务功能，建设魏井邻里中心和6个城市之家。打造智能信用、智能交通、智能城管、智能健康、智能人社，加快城市核心区、古黄河风光带等重点区域智能城市应用系统建设，增强市民“智能”感受度。提升繁荣度。整合古黄河滨水核心区、幸福路商圈资源，打造市级地标式西湖路商业中心。丰富宿城新区、宿豫区商圈业态，提升“一主两副”商圈能级。扎实推进高铁商务区建设，迎接高铁时代的到来。大力发展月光经济、楼宇经济、总部经济，办好“嗨在宿迁”消费促进系列活动，推动消费扩能升级，全面增强中心城市集聚力。

　　打造中国酒都。充分发挥千古酒乡的品牌优势，把酒韵、酒香、酒品融入城市肌理和城市文化。推动酒文化与城市设计融合。加快建设一批独具酒韵的城市建筑、城市小品，把洋河新区打造成酒文化的核心区。推动酒文化与城市产业融合。培育“酒+旅游”“酒+文化”“酒+商业”等关联产业，开发适合城市新阶层、新群体、新导向的新产品，促进酒文化和城市产业协调发展。推动酒文化与城市文化融合。以酒为脉，深入挖掘酒文化内涵，命名一批城市标识，开展一批文艺创作，举办一批时尚活动，全面打响宿迁酒都品牌。

　　打造旅游名城。坚持以全域视角谋划旅游业发展，着力建设旅游目的地城市。推进项目建设。深入推动洪泽湖湿地公园、三台山国家森林公园5A级景区创建，力争实现零的突破。加快推进骆马湖及古黄河水上游、项王故里等龙头型项目建设，提升旅游产业竞争力。加强产品开发。围绕旅游“六要素”，推进地方美食名吃、旅游商品开发，加快宾馆饭店、文化娱乐场所、旅游集散中心等建设，增强旅游配套功能。策划精品线路。串联三台山国家森林公园、项王故里、洪泽湖湿地公园、成子湖旅游度假区等景区，精心策划亲水游、文化游、乡村游等旅游线路，推动“一日游”向“多日游”“度假游”转变。强化品牌推广。高质量办好衲田花海音乐季、“国际生态四项公开赛”、“心宿情迁”四季游等活动，推动旅游宣传营销由知晓型向品牌型跃升。

　　打造电商名城。强化规划引领和政策支持，进一步做大做强电商产业，着力打造宿迁城市新名片。培育电商主体。积极招引知名电商企业区域总部落户宿迁，支持电商企业发展壮大，加快形成大小配套、结构合理的产业集群，新增网络销售2000万元以上电商企业10家。加强载体建设。进一步放大宿迁电子商务产业园区等平台支撑效应，加快京东云小镇、京东智慧城、京东物流二期等项目建设。深化省级跨境电商发展示范城市建设，促进跨境电商贸易便利化、运作阳光化，打造跨境电商贸易产业集聚基地。促进融合发展。加快发展智慧物流、互联网金融、大数据和电子商务服务业，推动电子商务城乡一体化发展，构建完善的电商产业生态链，全市电商交易额突破1500亿元。

　　各县城要找准发展定位，提升城市品质，塑造品牌形象。沭阳县围绕建设花木之乡、花园城市，持续擦亮工业百强县品牌，推动城市精致建设；泗阳县围绕建设平原林海、最美县城，以创建全国文明城市为抓手，加快提升城市品质内涵；泗洪县围绕建设湿地之都、水韵城市，按照“精品、特色、活力”的城市发展定位，着力打造魅力水城。

　　（四）聚力改善住房条件，深入推进乡村振兴。坚持“三农”优先发展，促进农业全面升级、农村全面进步、农民全面发展。

　　着力推动镇村布局优化。加快构建“1+3+50+N”城乡发展空间格局，有序引导农民进城入镇。做强重点镇。按照小城市建设标准，打造一批镇区集聚5-10万人、具有综合性功能的重点镇，各县区在优化提升省级重点镇基础上，启动1-2个“小城市”建设。做优特色镇。按照工贸型、农贸型、旅游型等发展方向，打造一批镇区集聚2-5万人、支撑农村经济发展的特色镇，各县区启动建设1-2个特色镇样板。做精新型农村社区。有序撤并偏远村、弱小村、空心村，打造一批集聚2000-3000人、承载留村居民集中居住的农村社区，全市启动建设110个左右新型农村社区。

　　积极稳妥推进农房建设。坚持规划引领，强化统筹调度，科学把握进度，在接续抓好44个集中居住项目的基础上，启动新建改造项目123个，建成市级示范项目15个以上。注重建设质量。严格把控建设标准，认真落实项目建设导则，精心精细施工，着力打造农房建设“精品工程”“样板工程”，确保群众住上安心房、放心房。加强特色塑造。挖掘乡村风貌特色和内涵，提炼宿迁民居特色元素，全面优化布局结构、整体风格、文化内涵以及立面设计、色彩搭配等，最大程度体现乡土特色风貌。优化功能配套。按照“五通八有十到位”标准，统筹做好管道管线、公共服务配套、园林景观等建设。坚持生活方式与生产方式同步转变，实现搬得出、稳得住、生活好。改善乡村环境。推进特色田园乡村建设，深入实施农村人居环境整治提升三年行动，整治县乡河道300条，建成绿化示范村、“三化”示范村30个。推进“厕所革命”，农村无害化卫生户厕普及率达93%以上。

　　大力发展现代农业。推动规模壮大。巩固提升粮食生产能力，推动藏粮于地、藏粮于技落实落地。加快特色产业培育，六大百亿级产业产值占比超过70%。新增生态高效农业25万亩、高标准农田44万亩，绿色优质农产品占比达50%。推动“三产”融合。加快农业产业园提档升级，实现农产品加工集中区建设县区全覆盖。发展休闲农业、创意农业，创建省级休闲观光农业精品村5个、主题创意农园5个。推动品牌做优。放大“宿有千香”农产品区域公用品牌溢出效应，积极培育农产品“三品一标”，新增绿色食品50个以上、地理标志登记农产品2个。推动主体培育。新培育家庭农场500个、农民合作示范社20家、市级以上龙头企业10家，创建农业产业化联合体10个以上，培训职业农民1.5万人次，促进小农户与现代农业有机衔接。

　　充分激发农业农村活力。深化集体建设用地改革。落实宅基地集体所有权，保障宅基地农户资格权，适度放活农房使用权和宅基地使用权。深化承包地改革。放活农村承包土地经营权，积极引导农民土地入股、参与经营，培育村级土地股份合作社示范社20家。深化产权制度改革。加快农村集体“三资”清产核资，力争70%的行政村完成股份经济合作制改革。实施振兴村集体经济三年行动计划，以县区为单位村级集体经济经营性收入增长7%以上。进一步完善农村集体产权交易市场体系建设，深化土地增减挂钩节余指标省域内交易试点工作。

　　（五）聚力打好“三大攻坚战”，着力补齐小康短板。坚持底线思维、问题导向，全力抓重点、补短板、强弱项。

　　打好防范化解重大风险攻坚战。落实“坚定、可控、有序、适度”要求，确保风险总体可控。全力防控金融风险。实施打击恶意逃废债务、非法集资等五大专项行动，突出抓好企业资金链、担保链“两链”风险管控化解，确保守住不发生区域性、系统性金融风险底线。稳步化解政府性债务风险。坚持“去存量、控增量”，严格落实债务风险防范化解工作计划，规范政府融资行为，推动国有平台公司市场化转型，实现政府性债务风险水平总体下降。

　　打好精准脱贫攻坚战。深入开展“精准脱贫冲刺年”活动，实现22.46万低收入人口和38个省定经济薄弱村基本脱贫，省级扶贫开发重点县全部退出。强化产业扶贫。因村制宜建设扶贫车间，各县区分别建设1-2个投资不低于2000万元、单体带动低收入人口100人以上的产业扶贫重大项目。强化项目扶贫。抓好交通、农田水利等配套项目建设，实施三大片区整体帮扶项目200个以上。大力推进农村道路安全生命防护工程，减少因交通事故而引发的贫困现象。强化政策扶贫。认真落实健康扶贫、教育扶贫等政策，推进医保、养老保险财政代缴，确保应保尽保。强化搬迁扶贫。引导低收入农户有序向城市、乡镇和新型农村社区集中，切实改善生产生活条件。

　　打好污染防治攻坚战。抓好环境污染整治。扎实推进中央和省环保督察“回头看”反馈问题整改，持续推进“263”专项行动，深入实施“2330”工程，推动生态环境质量不断改善。加强挥发性有机物、城市扬尘、“散乱污”企业、化工企业“四个一批”、机动车船污染等专项整治，实现PM2.5平均浓度持续下降、空气质量优良天数比例持续上升。深入落实“河（湖）长制”，大力实施农村河道清流连通工程，基本消除城市黑臭水体，国考和省考断面水质持续稳定达标，全市优Ⅲ类水体比例达73%以上。抓好基础设施配套。推进城镇村三级污水收处设施建设，实现乡镇污水处理管网全覆盖、农村污水处理设施覆盖率达75%以上。推行垃圾分类处理，中心城市、三县建成区生活垃圾分类投放设施覆盖率分别达70%、60%以上。抓好生态修复提升。加快百万亩生态林建设，新增生态林9.8万亩、更新改造杨树25.36万亩。深入开展洪泽湖、骆马湖“退渔还湖”工程，压缩围网养殖6万亩。

　　（六）聚力优化营商环境，有效释放发展活力。围绕更好地激发微观主体活力，积极推动理念提升、服务提升、路径提升、效率提升，着力打造全省营商环境最优城市。

　　构建公平竞争的市场环境。持续深化简政放权。认真落实中央和省各项改革部署，进一步深化行政审批制度改革，全面推行“证照分离”“证照联办”，在企业开办、不动产登记和施工许可领域实现“2345”改革目标。推进“不见面审批”标准化建设，尽快实现“一网通办”。开展“减证便民”行动，进一步减少备案、确认、告知等环节和手续。减少市场准入限制。逐步消除各类显性或隐性门槛，鼓励民间资本更大范围、更深层次参与经济建设。打造良好市场秩序。深化综合行政执法体制改革，健全监管机制。开展重点领域不正当竞争行为集中整治，严厉打击串标、转包、资质挂靠等行为，切实净化公共资源交易市场环境。加大对失信行为的约束惩戒力度，着力打造“信用宿迁”。

　　营造优质高效的政务服务。优化帮办服务工作机制。认真贯彻落实中央和省民营企业座谈会精神，坦荡真诚同企业家接触交往，真心实意为企业解忧纾困。拓展帮办服务内涵，完善帮办服务机制，探索建立企业网络帮办服务平台，打响“宿迁帮办”品牌。完善营商环境监测评价体系，以评促改、以改促优，推动营商环境持续改善。加快历史遗留问题化解。开辟证照办理绿色通道，着力解决企业有房无证、有地无权等问题。扎实做好环保关停企业技改复产工作，帮助企业恢复合法合规生产。

　　完善实惠管用的政策支持。认真贯彻好中央和省有关惠企政策，出台支持民营经济发展政策意见，助推实体经济健康稳定发展。围绕更多降低企业成本，加大涉企行政事业性收费清理力度，进一步降低企业用水、用气、用热、物流等成本，确保工商业用水、用气价格全省最低。围绕更优保障企业生产，优化水电气热等生产要素供给，努力保障企业生产需求。加快推广信用贷款、应收账款融资等模式，健全完善转续贷应急机制和担保体系，扩大制造业贷款投放；鼓励社会资本设立创业、风险、产业、并购等股权投资基金，支持企业利用债券融资，年内新增企业直接融资200亿元。加快建设市外劳务合作基地，组织开展“民营企业招聘周”、人才科技恳谈会等活动，扩大人才“购房券”补贴覆盖面，不断优化人力资源供给。围绕更实支持企业转型，制定出台推进科技创新、促进实体经济高质量发展等政策意见，充分发挥产业基金作用，分类引导企业加强科技研发、技术升级、产品更新，鼓励支持企业做大规模、做优质态。

　　打造安定有序的法治保障。严厉打击扰乱市场秩序、侵犯知识产权、制售假冒伪劣商品等违法行为，引导各类市场主体依法合规经营。健全企业救治和退出机制，促进生产要素优化组合和产业转型升级。加快完善企业维权“绿色通道”，依法保护企业家人身权和财产权，营造尊重企业家浓厚氛围。

　　（七）聚力促进社会和谐，持续增进群众福祉。始终把人民群众对美好生活的向往作为奋斗目标，全力做好底线民生、基本民生和质量民生，不断增强群众的获得感、幸福感。

　　织密富民增收网。推进创业富民。实施职业技能培训“三年行动计划”，建设返乡创业园、创业示范基地，引导返乡人员、退伍军人、大学生等群体投身创业，年内新增创业带动就业人数不低于6.3万人。推进就业富民。实施更加积极的就业政策，扎实开展“家门口就业”工程，完善覆盖城乡的公共就业服务体系。

　　织密公共服务网。促进教育优质均衡。大力实施中心城区学校建设三年攻坚和基础教育高质量发展三年行动计划，打造教育高地。加快全市30所中小学幼儿园建设，着力缓解“大班额”“就学难”等问题。规范中小学办学行为和校外培训机构，切实减轻学生过重课业负担。合理配置普通高中和中等职业教育资源。积极发展高等教育，推进产教融合发展。创新教师补充机制，努力缓解教师缺口问题。增强医疗服务能力。进一步完善医疗卫生服务体系，各县区规划建设1-2个、全市建设一批区域医疗卫生中心。优化运营管理，加快提升市第一人民医院医疗服务水平。支持社会办医高质量发展，年内市人民医院创成三级甲等综合医院。深入推进分级诊疗和双向转诊制度，抓好市县乡村医联体信息化建设，促进优质医疗资源重心有序下移。强化卫生人才招引和培训，着力建强医卫人才队伍。优化区域供水保障。实施区域供水完善提升工程，全面更新改造供水老旧管网，保障农村地区供水水质合格、水压稳定。完善集中式饮用水水源地长效管护机制，启动银控二水厂四期工程。提高养老服务供给。实施国家级居家和社区养老服务改革试点，居家养老服务老人占比达7%以上。支持社会资本兴建养老机构，加快推进“医养融合”，更好满足多样化养老需求。繁荣发展文体事业。推进基层文化阵地建设，开展惠民演出1000场以上。完善“十分钟健身圈”功能，广泛开展全民健身活动。全力办好市五运会。

　　织密社会保障网。实施全民参保计划和社保扩面“幸福行动”，落实城乡低保、农村“五保”和孤儿供养标准自然增长机制，切实兜好保障底线。统筹推进社会福利、慈善事业、优抚安置工作，加快构建“一门受理、协同办理”社会大救助机制。坚持“房子是用来住的、不是用来炒的”，加快定销商品房建设，完善住房市场体系和住房保障体系，让更多群众尽快改善住房条件。

　　织密社会安全网。深入推进网格化社会治理，构筑共建共治共享格局。着力预防和化解社会矛盾，维护好社会稳定大局。深化“平安宿迁”建设，深入推进扫黑除恶专项斗争，加快构建立体化现代化治安防控体系。严格落实安全生产责任制，持续加强危化品、消防、道路交通、建筑施工等行业领域安全生产监管，确保安全生产形势稳定可控。开展食品药品系列专项整治，筑牢食药安全底线。加强和改进信访工作，依法解决群众合理诉求。

全力建设人民满意政府

　　新一年、新气象，新征程、新作为。我们将牢固树立“四个意识”，切实增强“四个自信”，在思想上政治上行动上始终同以习近平同志为核心的党中央保持高度一致，秉持全心全意为人民服务的宗旨，不断提升政府施政能力和服务水平。

　　建设更加诚信法治的政府。坚持把诚信作为施政准则，以政府诚信带动社会诚信建设，不断增强政府公信力。切实加强依法行政，将政府活动全面纳入法治轨道。严格遵循“三重一大”事项集体决策等制度，不断健全政府科学决策、民主决策、依法决策机制。依法接受市人大及其常委会的监督，主动接受市政协的民主监督，重视司法、舆论、社会监督，进一步加强审计监督。加快推进政府信息公开标准化建设，始终让权力在阳光下运行。

　　建设更加开拓创新的政府。始终坚持用习近平新时代中国特色社会主义思想武装头脑，切实增强“八个本领”，不断提升研判大势、谋划工作、推动发展的能力和水平。牢固树立创新意识，推动思想大解放、改革再出发，勇闯新路、敢于实践，不断增强政府工作的开拓性和创造力。坚持系统化、市场化思维，积极改进组织和领导经济工作方式，恪守政府行动边界，更多发挥市场机制作用，不断提高资源配置效率。

　　建设更加担当有为的政府。坚持真抓实干，积极弘扬“三真”精神，大兴求真务实之风，说实话、谋实事、出实招、求实效。注重高质高效，大力倡导“马上办”作风，第一时间执行好中央、省和市委决策部署，第一时间回应群众反映强烈的问题。大力发扬苦干实干作风，遇到矛盾不回避，困难面前不退缩，努力以勤政指数赢得人民群众的满意指数、幸福指数。

　　建设更加风清气正的政府。严格执行廉洁从政各项规定，全面落实“一岗双责”，紧盯重点领域和关键环节，深化廉政风险防控。大力整治庸政懒政怠政和不作为、乱作为，推动政府工作提能提速提效。坚决贯彻中央八项规定精神和省、市委具体要求，全面加强政府机关作风建设，驰而不息纠“四风”、正作风，弘扬正气，凝聚正能量，牢固树立政府在人民群众中的良好形象。

　　各位代表、同志们！当前，我市正处在全面建设小康社会的关键期和攻坚期，任务艰巨、使命光荣、责任重大。让我们更加紧密地团结在以习近平同志为核心的党中央周围，在市委的坚强领导下，在市人大、市政协的监督支持下，在宿迁全体干部群众的共同努力下，同心同德、群策群力，锐意进取、奋力攻坚，为加快建成“强富美高”全面小康新宿迁而努力奋斗！
